# Neural Conversion and Patterning of Human Pluripotent Stem Cells: A Developmental Perspective

**DOI:** 10.1155/2016/8291260

**Published:** 2016-03-16

**Authors:** Alexandra Zirra, Sarah Wiethoff, Rickie Patani

**Affiliations:** ^1^Department of Molecular Neuroscience, UCL Institute of Neurology, Queen Square, London WC1N 3BG, UK; ^2^Center for Neurology and Hertie Institute for Clinical Brain Research, Eberhard-Karls-University, 72076 Tübingen, Germany; ^3^National Hospital for Neurology and Neurosurgery, UCL Institute of Neurology, 33 Queen Square, London WC1N 3BG, UK; ^4^Department of Clinical Neurosciences, University of Cambridge, Cambridge CB2 0QQ, UK; ^5^Euan MacDonald Centre for MND, University of Edinburgh, Edinburgh EH16 4SB, UK

## Abstract

Since the reprogramming of adult human terminally differentiated somatic cells into induced pluripotent stem cells (hiPSCs) became a reality in 2007, only eight years have passed. Yet over this relatively short period, myriad experiments have revolutionized previous stem cell dogmata. The tremendous promise of hiPSC technology for regenerative medicine has fuelled rising expectations from both the public and scientific communities alike. In order to effectively harness hiPSCs to uncover fundamental mechanisms of disease, it is imperative to first understand the developmental neurobiology underpinning their lineage restriction choices in order to predictably manipulate cell fate to desired derivatives. Significant progress in developmental biology provides an invaluable resource for rationalising directed differentiation of hiPSCs to cellular derivatives of the nervous system. In this paper we begin by reviewing core developmental concepts underlying neural induction in order to provide context for how such insights have guided reductionist in vitro models of neural conversion from hiPSCs. We then discuss early factors relevant in neural patterning, again drawing upon crucial knowledge gained from developmental neurobiological studies. We conclude by discussing open questions relating to these concepts and how their resolution might serve to strengthen the promise of pluripotent stem cells in regenerative medicine.

## 1. The Developmental Origins of the Nervous System: An Overview 

**Figure 1:**
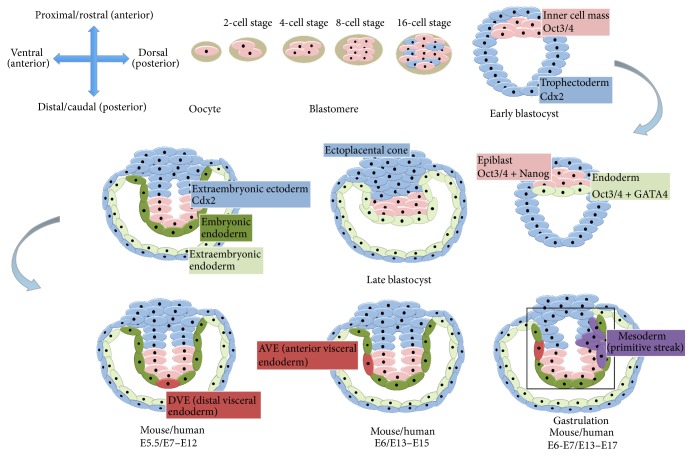
Developmental stages of mouse embryo. First row (left to right), from the secondary oocyte the blastomere develops (2-cell, 4-cell, 8-cell, and 16-cell stages) to give rise to the early blastocyst formed of trophectoderm (cells that express Cdx2) and inner cell mass cells (that express Oct3/4). Later, the inner cell mass gives rise to the epiblast (cells that express Oct3/4 and Nanog) and endoderm (expressing Oct3/4 and GATA4). Second row (right to left), in the late mouse blastocyst Cdx2 positive cells give rise to the extraembryonic ectoderm and ectoplacental cone. At the same time the endoderm divides into an embryonic endoderm and an extraembryonic endoderm. The epiblast and the extraembryonic ectoderm form a cavity lined by embryonic endoderm. From the embryonic endoderm the distal visceral endoderm is formed (DVE). Third row (left to right), the DVE migrates proximally and will be known as the anterior visceral endoderm (AVE). The final image (third row, right) shows the development of the primitive streak (mesodermal cells) at the opposite (posterior) pole from the AVE. N.B. There are 2 different types of endoderm called extraembryonic and embryonic; these differ in their potency and give rise to distinct cellular derivatives. All timelines are given for mouse and human embryonic development.

**Figure 2:**
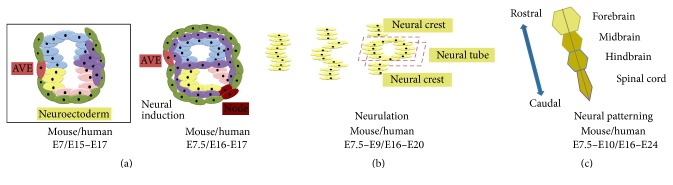
Neural induction, neurulation, and neural patterning overview. (a) Neural induction: neuroectoderm (neural plate) differentiation happens under the influence of the AVE. The mesodermal cells start migrating in all directions and envelop the embryo between the endoderm and the ectoderm. At the distal pole of the embryo the node develops, to further act as the “trunk organiser.” (b) Neurulation: from the neural plate, cells start to proliferate and invaginate in order to form the neural tube and neural crest which derives from the dorsomedial borders of the neural folds. (c) Neural patterning: cells from the neural tube start to differentiate into precursors for forebrain, midbrain, hindbrain, and spinal cord according to a rostrocaudal axis. All timelines are given for mouse and human embryonic development.

The process of neurodevelopment is spatiotemporally regulated and necessitates sequential, progressive restrictions in cell fate. Although some interspecies differences in both cytoarchitecture and molecular machinery do exist between mouse and man, rodent models have illuminated key underlying mechanisms of lineage restriction to a variety of cell types. These insights have provided invaluable guidance for the predictable manipulation of human pluripotent stem cells (hPSCs) into myriad cell fates. From the point of fertilisation of the secondary oocyte, cells commence asymmetric division and sequentially give rise to the 2-, 4-, and then 8-cell stage blastomere, which subsequently develops into the blastocyst (Figure 1). Oct3/4 serves to maintain pluripotency in the inner cell mass (ICM) of the blastocyst. Although interspecies differences in cell-type specific factors exist, ultimately and following implantation and gastrulation, 3 distinct germ layers emerge: endoderm (which forms the lining of internal organs), mesoderm (which gives rise to bone, muscle, and vasculature), and ectoderm (from which results the nervous system and skin). Figures 1 and 2(a) describe developmental processes involved in specification of the 3 germ layers. During gastrulation, this 3-layered structure undergoes progressive and stereotyped morphological transformations. The mesoderm and endoderm invaginate inwards and the ectoderm forms an epithelial sheet which ensheathes a central cavity. The region of the ectoderm surrounding the neural plate becomes epidermis (Figure 2(a)). An important aspect of embryogenesis is the assignment of developmental axes. “Anterior-posterior” can be used to refer to the proximal-distal axis, which is based on proximity to the future placenta (in the early blastocyst the proximal pole is represented by the ectoplacental cone as depicted in Figure 1). Later, the proximal-distal axis will become the future rostrocaudal axis in vertebrates. However, the term “anterior-posterior axis” can also sometimes refer to the dorsoventral axis in the adult state, a distinction that is primarily based on position of the abdomen (ventral) as opposed to the back/spinal column (dorsal). Therefore, for ease of reference this review will use the terms rostrocaudal (“R-C”) and dorsoventral (“D-V”) axes.

Three principal events characterise early neurodevelopment. First, the process of* neural induction* specifies a region of the embryonic ectoderm to form the neural plate (Figure 2(a) [1]). Second, a process termed* neurulation* occurs through serial morphological transformations to give rise to the neural tube (Figure 2(b); [2]). This process consequently imparts further histological architecture to the developing neuraxis. Third, the neural tube is divided into functionally and spatially distinct regions by a programme of inductive interactions called* neural patterning* (Figure 2(c) [3]). In humans, neurulation occurs at 21 days after conception and depends on a precise sequence of changes in the three-dimensional shape of individual cells including changes in cell-cell adhesion. Specific gene expression profiles are controlled by neuraxial position and local extrinsic morphogenetic instruction. Gastrulation leads to the formation of the notochord, a distinct cylinder of mesodermal cells extending along the midline. Ectoderm lies adjacent to the notochord, from which it receives inductive signals to form neuroectoderm. Neuroepithelium of the neural plate then undergoes complex morphogenetic movements involving cell division, morphological changes, and migration to permit neural tube formation. Following neural tube closure, the dorsomedial borders of the neural folds become neural crest derivatives. Cell movements at this stage are critical in producing different neuraxial regions. For example, in the ventral midline of the neural tube, cells become a specialized region called the floor plate (Figure 4(d)). A large variety of distinct neuronal subtypes are generated during mammalian neurodevelopment. This diversity is an absolute prerequisite for the establishment of functional neuronal circuits.

In summary, the consecutive steps of neurodevelopment include neural induction from embryonic ectoderm, patterning along rostrocaudal (R-C), and dorsoventral (D-V) axes (allowing regionally determined functional heterogeneity) and subsequently terminal differentiation into diverse postmitotic neuronal subtypes [2]. Such insights from developmental neurobiology provide a conceptual framework for the directed differentiation of hPSCs and allow experimental interrogation of the molecular “logic” of neuronal subtype diversification [4]. Taken together with the understanding that region, and/or subtype, specific degeneration of neurons underpin the majority of neurodegenerative diseases, these facts provide a compelling rationale to predictably manipulate the cell fate of hPSCs in order to generate clinically relevant populations of region specific neurons and glia for further study [5].

## 2. Neural Induction 

**Figure 3:**
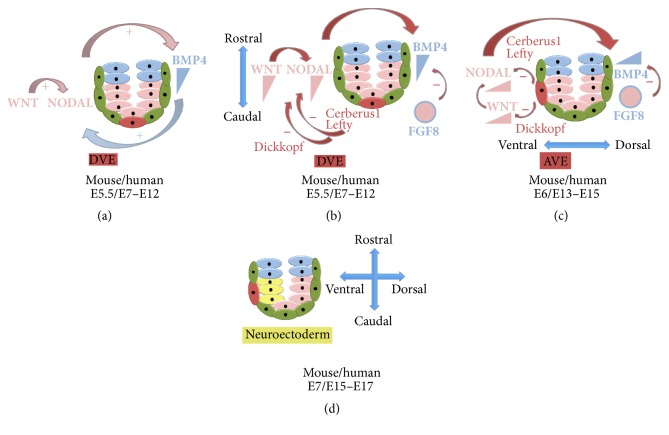
Molecular pathways in neural induction. (a) The epiblast (depicted in pink) expresses Nodal. The epiblast through Nodal stimulates (pink arrow) the expression of BMP4 (depicted in blue) in the extraembryonic ectoderm (blue cells). The extraembryonic ectoderm, by the action of BMP4, stimulates (blue arrow) the WNT (depicted in pink) pathway in the epiblast that in turn further activates (pink arrow) Nodal expression. Thus, there is a positive feedback loop between Nodal, BMP, and WNT. Colour scheme: arrows corresponds to the related tissue/morphogen. (b) The DVE (depicted in red) expresses Cerberus1 and Lefty (also depicted in red) to inhibit Nodal expression, therefore downregulating Nodal in its proximity. It also expresses Dickkopf (depicted in red), a protein that inhibits WNT3 signals close to the DVE. Downregulating Nodal and WNT also inhibits BMP4 expression close to the DVE. Thus, there is a gradient of Nodal, WNT, and BMP with a high expression rostrally and low expression caudally. FGF8 (pink and blue), expressed both in the epiblast (pink) and extraembryonic ectoderm (blue), also inhibits BMP4 contributing to the gradient. Colour scheme: arrows corresponds to the secreted inhibitory molecules/tissue source (DVE). They show the consequence of the negative feedback that creates the morphogen gradients in the R-C axis. (c) The DVE migrates into the AVE and the gradients are thus remodelled with low Nodal, WNT, and BMP expression ventrally and high dorsally. (d) Due to these gradients the neuroectoderm is formed at the ventral pole of the epiblast. Colour scheme: arrows corresponds to the secreted inhibitory molecules/tissue source (AVE). They show the consequence of the negative feedback that creates the morphogen gradients in the D-V axis. All timelines are given for mouse and human embryonic development.

The first mechanistic insights into neural induction originate from seminal experiments by Spemann and Mangold in the early part of the twentieth century. In these studies, dorsal mesoderm was transplanted into the ventral embryo and generated a secondary host-derived neural tube. The graft itself was found to contribute to secondary mesodermal structures including the notochord, while the neural tissue was host-derived. The ability of the dorsal blastopore lip to reprogram surrounding tissues when transplanted ectopically justifies its designation as “organiser tissue.” Equivalent organiser regions in other vertebrates were subsequently discovered by the elegant work of Waddington in the 1930s, including “Hensen's node” in birds and mammals (Figure 2(a)). Organiser tissue's capacity to precipitate ectopic neural induction* interspecies* suggests evolutionary conservation of underlying mechanisms. The notion of inductive signals orchestrating the process of neural induction has become widely accepted. Accumulating evidence suggests a spatiotemporal interdependence of several signalling pathways in neural induction, which somewhat challenges the concept of organiser tissue. The molecular pathways underlying neural induction remained elusive until the 1990s, when* Xenopus* studies first reported that transient dissociation of gastrula-stage animal caps into single cells resulted in neural fate acquisition and that misexpression of a dominant-negative Activin receptor, since being discovered to inhibit multiple transforming growth factor (TGF*β*-) related factors, ectopically generated neural tissue at the expense of mesoderm specification. These studies suggest that neural induction may occur through a “de-repression” strategy (i.e., the removal of an inhibitory signal).  Figure 3 depicts the relevant pathways in this process.

### 2.1. The Role of TGF-*β* Signalling Superfamily Members in Neural Induction

The molecular machinery of TGF-*β* signalling is relatively well understood: ligand binding causes receptor dimerization and initiates a signal transduction pathway and activates a family of cytoplasmic proteins, the Smads, by phosphorylation. Eight Smad proteins are encoded in the human genome, although only five of these (Smad 1, Smad 2, Smad 3, Smad 5, and Smad 8) act as substrates for the TGF receptor family; these are commonly referred to as “receptor-regulated Smads,” or just “RSmads.” Broadly, the TGF-*β* signalling superfamily encompasses both the Activin/Nodal and bone morphogenetic protein (BMP) signalling pathways [6]. The substrates for BMP signalling are Smads 1, 5, and 8, while the Activin/Nodal receptors activate Smads 2 and 3. Co-Smad (Smad 4) functions as a common partner for all RSmads, whereas Smad 6 and Smad 7 are inhibitory. Smad/Smad 4 complexes translocate to the nucleus and activate gene expression.

#### 2.1.1. BMP Antagonism

In the early 1990s Noggin, Follistatin, and Chordin were identified as genes encoding proteins with neuralizing activity that were expressed in organiser tissue. These proteins are inhibitors of BMP signalling, with a particular bias towards antagonising BMP4, an inhibitor of neural fate. BMP4 is expressed widely at the onset of gastrulation (Figure 3(a)) but is subsequently downregulated in the neural plate following the emergence of the organiser region (Figure 2(b)). Blockade of BMP signalling leads to an expanded neural plate in whole embryos, while mice with null mutations in BMP antagonists (such as Noggin and Chordin) show a significantly reduced brain size [1]. The wider roles of BMP pathway in embryo development are comprehensively reviewed elsewhere [7].

These facts, taken together, allow a simple molecular pathway for neural induction to be considered: the extraembryonic ectoderm produces BMPs to promote epidermal differentiation, while neural inducing regions (organiser tissues) antagonize BMPs to permit neural induction (Figures 3(a)–3(d)). This can be achieved by blocking BMP mRNA at the pregastrula stage by Fibroblast Growth Factor (FGF). Alternatively, the BMP protein can be antagonised at the gastrula stage by aforementioned factors secreted from organiser regions. Against this background, the “default model” of neural induction was formulated, hypothesizing that gastrula-stage ectodermal cells have an autonomous predilection to differentiate into neural tissue and that this process is inhibited by BMPs. In contrast to this model, subsequent studies have demonstrated that organiser tissue/BMP antagonism can be dispensable for neural induction, suggesting that additional mechanisms/signalling pathways merit consideration in this review, given their potential significance in informing strategies for neural conversion of hPSCs [1, 8, 9].

#### 2.1.2. Activin/Nodal Antagonism

A significant majority of studies have focused on the role of BMP inhibition in neural induction during vertebrate development. However, the importance of other members of the TGF-*β* superfamily, including Nodal, is also well established [10]. Nodal acts as an inhibitor of neural induction [11], while Nodal knockout embryos show increased neuroectoderm specification [12]. A role for Nodal inhibition in neural induction from mouse and human embryonic stem cells (ESCs) is well established, both alone [13–15] and combinatorially with BMP antagonism [16]. Nodal is expressed throughout the epiblast (Figure 3(a)) and inhibitors of this pathway have been identified in the DVE/AVE [17], which play crucial regulatory roles both in neural induction and in repositioning morphogen gradients between the R-C and D-V axes (Figures 3(b) and 3(c)). Against this background, we and others have utilised Nodal antagonism alone to achieve neural specification from hPSCs in suspension culture [14–16, 18], although the most widely adopted approach to neural conversion from hPSCs is termed dual-Smad inhibition and utilises both Nodal and BMP4 antagonists in combination [16].

### 2.2. Other Factors Implicated in Neural Induction

#### 2.2.1. Fibroblast Growth Factors (FGFs)

FGFs are a diverse collection of secreted diffusible glycoproteins that act by binding with differential affinity to four classes of extracellular receptor (FGFR 1–4). The precise role of FGF signalling in neural induction remains controversial, but studies collectively suggest an early function to promote competence for neural conversion and later functions in transcriptional antagonism of BMP. Another important member of the FGF family, FGF8, is expressed in the mouse embryo in the extraembryonic ectoderm and the epiblast before and during gastrulation (Figures 3(b) and 3(c)). FGF8 activates calcineurin, which dephosphorylates Smad 1/5, the main components of the BMP4 pathway [19]. Thus, FGF8 can inhibit BMP4 signalling leading to neural induction. This finding further supports the complexity of neural induction and somewhat challenges the previous “default” model. Human PSC biology has also contributed to understanding the relevance of FGF in neural induction, with some studies demonstrating that FGF withdrawal or antagonism (together with Nodal and BMP4 antagonism) facilitates neural conversion [20–22], and others suggesting that FGF has neural inducing capacity [23–26]. These seemingly contradictory findings can be at least partially reconciled through recognition that different culture conditions were employed in each of these studies (e.g., monolayer versus suspension culture; different programmes of coadministered extrinsic signals), which may alter the influence of FGF on neural induction in a context-dependent fashion.

#### 2.2.2. WNT Signalling

WNTs are secreted glycoproteins responsible for establishment of the dorsoventral axis of the embryo, a direct consequence of which is the acquisition of neural identity. Administration of mRNA encoding WNTs (or their effectors) into the animal hemisphere of one-cell embryos by injection generates ectopic neural tissue. WNT signalling is itself activated by BMP4 and implicated in a Nodal positive feedback loop [27] (Figure 3(a)). The AVE secretes Dickkopf, a WNT pathway antagonist contributing initially to the R-C, and later the D-V, Nodal gradient (Figures 3(b) and 3(c)). However, WNT3 activation does not impair neural induction in mouse embryos [28], mESCs [29], and hiPSCs [30]. An extra layer of complexity is added by the different ways in which WNT can act throughout development, the canonical *β*-catenin pathway (to promote proliferation), or the noncanonical JNK pathway (to promote neuronal differentiation) in an FGF2-dependent manner [31].

These findings collectively suggest that neuroectoderm specification is likely more complex than the “default” (BMP4 inhibition) or “organiser” (combined BMP4, WNT3, and Nodal inhibition) models might suggest. The effects of each relevant signalling pathway are temporally regulated and determined by developmental context, justifying their systematic investigation (both individually and combinatorially) in the neural conversion of hPSCs [26].

## 3. Neural Patterning: An Overview

**Figure 4:**
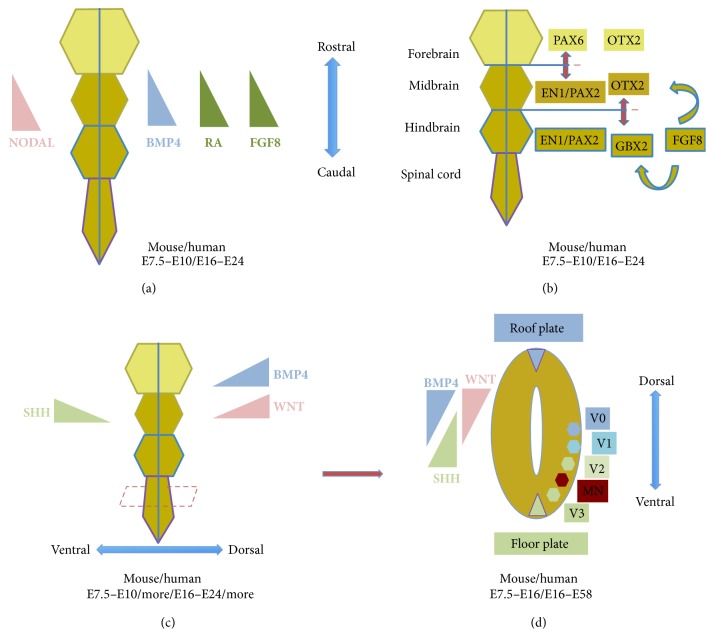
Neural patterning. (a) Rostrocaudal gradients of Nodal, BMP4, RA (retinoic acid), and FGF8 important in rostrocaudal patterning. (b) The interplay between different factors encoding forebrain (PAX6 and OTX2), midbrain (PAX6, OTX2, and EN1/PAX2), and hindbrain (EN1/PAX2, GBX2, and FGF8). The forebrain-midbrain barrier is defined by the mutually exclusive expression of PAX6 (forebrain) and EN1/PAX2 (midbrain), while the midbrain-hindbrain boundary by OTX2 (midbrain) and GBX2 (hindbrain). OTX2 and GBX2 are regulated by FGF8 expression. (c) Dorsoventral patterning with dorsal gradients for BMP4 and WNT and with a ventral gradient of SHH (Sonic hedgehog). (d) Transverse section through the neural tube depicting various neurons specified by the gradient of SHH from the floor plate and the BMP4 and WNT from the roof plate: V0–3: interneurons and MN: motor neurons.

Once specified, the neuroectoderm is subsequently regionalized along the R-C axis of the embryonic body (Figures 2(c) and 4(a)). Organiser regions can be divided into those that are involved in generating rostral versus caudal structures in the neuraxis [32]. More specifically, following gastrulation the head organiser tissue lies under the prechordal neural plate (anterior neurectoderm), whereas tail organiser tissue becomes notochord and somites and lies beneath the epichordal neural plate (posterior neurectoderm). Interestingly, there is evidence that during neural induction in mESCs, WNT and FGF signalling promote neuromesodermal precursors, a population of cells that gives rise to spinal cord neurons and paraxial mesoderm [29]. Signals that inhibit BMPs (e.g., Noggin) and WNTs (e.g., Dickkopf) stimulate production of the prechordal plate, insights which have again guided ontogeny recapitulating hPSC differentiation protocols [33].

The precise timing and mechanisms of neuraxial patterning remain unresolved. A popular model is that neural induction initially specifies rostral precursors, upon which caudalising signals subsequently respecify positional identity in a progressive and stereotyped manner to establish subdivisions of the posterior neuraxis. Some of the signalling pathways implicated in neural induction also appear to play key roles in early R-C and D-V patterning at later stages [10]; they establish a matrix of positional cues (Figures 4(a) and 4(c)), which in turn influence precursor cell fate specification through graded concentrations of morphogenetic signals. In broad terms, the anterior neuroectoderm generates the forebrain, and the posterior neuroectoderm gives rise to the midbrain, hindbrain, and spinal cord [32]. The D-V signalling pathways have more pertinent roles in generating neural cell-type diversity within each of the aforementioned R-C subdivisions (Figure 4(c)). It is noteworthy that other mechanisms, such as local signals between developing neurons, also contribute to the full ensemble of neuronal subtypes. Figure 4 summarizes some of the relevant concepts here, which are explained in further detail below.

### 3.1. Early Patterning in the R-C Axis

Evidence from animal studies suggests that spatially and functionally distinct cell populations organise development of head and trunk structures [32]. The head organiser tissue is located in the AVE and the trunk organiser in the node and anterior primitive streak (Figure 2(a)). A wealth of evidence implicates BMP antagonism in forebrain development (Figure 4(a)). Indeed, neural conversion strategies utilising BMP antagonism in hPSCs generally report forebrain precursor specification [16, 23, 34, 35].

Studies using a range of approaches have shown that AVE is necessary for normal forebrain development with Nodal signalling being critical in this process [1]. Collectively, these studies suggest that partial reduction of Nodal signalling primarily affects specification of the prechordal mesendoderm, which is necessary for antagonising caudalising signals and thus perturbs forebrain development. Therefore, Nodal signalling is necessary for proper R-C patterning of the neuroectoderm (Figure 4(a)). Smad 2 and Smad 3 are requisite intracellular effectors of Nodal signals. Previous reports implicate Smad 2/3 in neural development; in mice, for example, Smad 2^+/−^ and Smad 3^−/−^ mutant embryos exhibit a miniaturized head-like structure [36]. In zebrafish, injection of mRNAs encoding dominant-negative Smad 2/3 mutants also results in a smaller head [37]. However, the precise roles of Smad 2/3 in neural induction and neuroectodermal patterning remain incompletely understood. Against this background and consistent with these findings, we and others have demonstrated that small molecule inhibition of Smad 2/3 imposes caudal regional identity on hPSC-derived neural precursors [15, 26].

A FGF signalling gradient operates along the R-C axis to induce the expression of paralogous Hox genes in the neural tube. Hox genes located at one end of the cluster (3′ end) are expressed more rostrally in response to low levels of FGF; conversely genes at the opposite end (5′ end) are expressed caudally in response to high levels of FGF (Figure 4(a)). Different Hox genes are consequently expressed at brachial (Hox4–Hox8), thoracic (Hox8-Hox9), and lumbar (Hox10–Hox13) levels of the neural tube [38]. The mechanisms by which a Hox-based transcriptional network choreographs these processes are now being systematically resolved [39]. These graded FGF signals regulate the primary Hox gene expression pattern before further superimposed cues refine subset-specific Hox expression. Rostrally, retinoic acid (RA) regulates Hox expression at cervical/brachial levels, in part by antagonising the FGF gradient (Figure 4(a)). More caudally, Gdf11 (also a member of the Tgf-*β* superfamily) plays an important role in Hox8–Hox10 gene expression at thoracic and lumbar neural tube regions [40].

### 3.2. Patterning in the D-V Axis

The D-V arrangement of neuraxial anatomy is closely correlated to functional organisation. This anatomical polarity is clearly evident in the spinal cord where motor neurons reside in the ventral horns and sensory neurons are positioned in dorsal root ganglia. In the rostral neuraxis, structures such as the basal ganglia (including the substantia nigra) are ventrally located, while the cerebral cortex is dorsally positioned. R-C and D-V patterning is carefully integrated in a highly stereotyped manner. Broadly, ventral regional specification requires activation of both the Nodal and Sonic hedgehog pathways with antagonism of BMP signalling. Over and beyond its role in R-C patterning, RA is required for intermediate zone specification within the D-V axis. Likewise, FGF also plays important roles in ventral domain specification. The major contributors to D-V axis formation are BMPs and WNTs dorsally, and Sonic hedgehog ventrally [41]. Distinct neuronal subtypes are generated through interaction of opposing D-V morphogenetic gradients, which form a matrix of “coordinates” that combinatorially encode discrete precursor domains in a stereotyped D-V array [2, 3]. In the neural tube, this developmental strategy underlies motor neurogenesis and ventral interneurogenesis (Figure 4(d)). Ventral neural patterning results from morphogens originating from the floor plate and the notochord. In the early 1990s, different labs cloned vertebrate homologues of the* Drosophila* gene hedgehog, which encode secreted signalling proteins. Sonic hedgehog (SHH) transpired as the ventrally secreted morphogen conferring D-V neural tube polarity (Figure 4(c)). It is now well established through a variety of gain- and loss-of-function studies in different species that SHH plays crucial and indispensible roles in specifying ventral cell types throughout the neuroectoderm [41]. SHH is first expressed in the notochord and later the floor plate, likely secondary to auto induction (Figure 4(d)). Its function is concentration-dependent and its major effector mechanism is repression of GLI3 transcription factor. Spinal motor neuron generation, for example, depends on two temporally distinct phases of SHH signalling: an early period where it ventralizes neural plate precursors and a late period where it promotes differentiation of these precursors into motor neurons, at which point there is a concentration-dependent specification of ventral precursors into motor neurons or interneurons (Figure 4(d)).

How is positional identity imposed on precursor cells? Several studies have implicated a group of factors, predominantly the homeodomain (HD) and basic helix-loop-helix (bHLH) transcription factors, as crucial regulators here. These are expressed in strictly organised arrays along the D-V axis of the neural tube. Individual proteins are designated as classes I or II by their response to SHH signalling. Class I proteins are repressed by SHH, thus defining their ventral limit of expression, while class II protein expression is induced by SHH and defines dorsal expression boundaries. Specifically in the context of spinal cord development, such cross-repressive interactions allow the establishment of five distinct ventral precursor domains, which in turn permit the specification of distinct neuronal subtypes. Gain- and loss-of-function experiments have further supported this putative mechanism across different species, where ectopic expression of HD proteins predictably changed the regional allocation of individual neuronal subtypes within the neural tube [38, 42]. A similar cross-repressive interaction between protein classes I and II also underlies the developmental “logic” of ventral spinal neurogenesis. The most ventral aspects of neural patterning (i.e., floor plate) require Nodal signalling, and FGF has also been broadly implicated in ventral patterning within the neuraxis [41].

SHH signalling does not appear to contribute to patterning in the dorsal neural tube. However, BMPs have similar and complementary roles in dorsal patterning of the neural tube and telencephalon (Figure 4(c)). These serve as the primary dorsal morphogenetic cues by establishing a high to low concentration from dorsal to ventral positions. In a similar fashion to SHH in the ventral neural tube, this BMP gradient enables distinct precursor domains to be defined, thus permitting the generation of diverse dorsal neuronal subtypes [43].

## 4. Directed Differentiation of hPSCs

**Figure 5:**
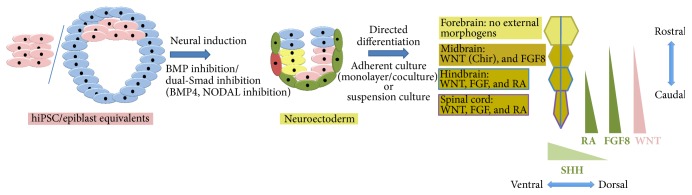
Methods for directed differentiation: hPSCs can be directed to undergo neural conversion by applying developmental principles of inhibiting BMP4 and/or Nodal. From neuroectoderm, differentiation of different neuraxial regions can be achieved by recapitulating developmental morphogenetic instruction: forebrain (default), midbrain (WNT, FGF8 activation), hindbrain (WNT, FGF8, and others, RA), and spinal cord (WNT, FGF8, and others, RA). These gradients are shown on the right of the figure for the rostrocaudal axis. Another important morphogenetic cue used in directed differentiation is SHH for its ventralising effect within the D-V axis. All timelines are given for mouse and human embryonic development.

These aforementioned developmental studies provide a conceptual framework to rationalise both neural induction strategies and bespoke programmes of morphogenetic cues for the directed differentiation of hPSCs to clinically relevant and region specific neurons (summarized in Figure 5 and Table 1).

### 4.1. Forebrain

“Default” neural conversion from hPSCs to forebrain neuronal subtypes has been demonstrated in a variety of systems including chemically defined suspension culture, not requiring extrinsic signals, as well as in an adherent culture method [16, 44, 45]. These studies began in 2007 with the discovery that a* selective Rho-associated kinase* (ROCK) inhibitor permits survival of dissociated hPSCs, thus allowing systematic manipulations to cell fate after dissociation [44]. A year later, the same lab again employed serum-free embryoid body-like (SFEB) culture but this time to recapitulate cell intrinsic and temporally regulated cortical laminar determination in vitro [45]. These and subsequent studies have confirmed cortical layer specific expression of different markers including Reelin in layer 1 (Cajal-Retzius neurons), TBR1 and CTIP2 in deep layers, and SATB2, BRN2, and CUX1 in superficial cortical layers [46]. Such default dorsal telencephalic differentiation strategies tend to give rise to predominantly glutamatergic, but also some GABAergic, neurons [47].

Prior to terminal differentiation, if specified dorsal telencephalic precursors are exposed to SHH and/or a WNT antagonist, they are ventralised to generate subpallial derivatives (i.e., of the lateral and medial ganglionic eminences; LGE and MGE, respectively). Upon terminal differentiation, these ventralised telencephalic cultures give rise to GABAergic projection neurons and interneurons. Clinically relevant cell types originate from the LGE (e.g., medium spiny projection GABAergic neurons, which are relevant to Huntington's disease and dystonia) and the MGE (e.g., basal forebrain cholinergic neurons relevant to Alzheimer's disease). Further sophistication can be added to the aforementioned directed differentiation strategies by carefully regulating SHH and WNT pathways (which orchestrate dorsoventral positional identity in this context). For example, a low concentration of SHH alone permits the specification of both LGE and MGE derivatives, whereas if a WNT antagonist is added to SHH, the more ventral MGE (i.e., NKX2.1 expressing) neurons are preferentially specified at the expense of LGE (i.e., GSX2, DLX, MEIS2, and ISLET1 expressing) neurons. Some elegant and ontogeny recapitulating strategies have been defined for the generation of authentic DARPP32 expressing medium spiny projection neurons [33, 47].

### 4.2. Midbrain

Differentiating hPSCs into midbrain dopaminergic neurons has maintained great enthusiasm likely owing to their potential to understand and treat Parkinson's disease. Although dopaminergic neurons exist throughout the nervous system, there is a region-specific functional heterogeneity that has been experimentally demonstrated by performing anisotopic implantation experiments [48]. Midbrain dopaminergic neurons are developmentally partitioned to three distinct nuclei: (i) the substantia nigra pars compacta (A9 group), which is primarily affected in Parkinson's disease, (ii) the ventral tegmental area (A10 group), and (iii) the retrorubral field (A8 group). Noting that hPSC-derived neural precursors have a default rostral (forebrain) and dorsal (cortical) identity, morphogen-guided positional respecification, or patterning, to the ventral mesencephalon is necessary for the differentiation of authentic midbrain dopaminergic neurons. Feeder-free and feeder-dependent differentiation approaches have both been employed to generate midbrain dopaminergic neurons from hPSCs. Feeder-dependent differentiation strategies have utilised mouse stromal cell lines (e.g., PA6), which, even though relatively easy to establish, carry the main disadvantage of being chemically undefined and animal-derived. From developmental in vivo studies, we are guided by the insight that FGF8 signalling leads to a cross-repressive interaction between Otx2 and Gbx2, defining the midbrain-hindbrain boundary (MHB; Figure 4(b)) and imparting rostrocaudal positional identity to precursors of the MHB [49]. Otx2 and Gbx2 control patterning in this region by regulating the expression of two morphogenetic cues, WNT1 in midbrain and FGF8 in the hindbrain. Furthermore, in combination with Otx2 expression, cross-repressive mechanisms between Pax6 and En1/Pax2 define boundaries of regional fate allocation to either forebrain or midbrain (Figure 4(b)).

Against this background, initial approaches to midbrain differentiation were based on FGF8 for R-C patterning to the region of the midbrain, and SHH for ventralization into dopaminergic neurons, although the yields were low (approx. 30%) using such strategies. Furthermore, subsequent studies have raised the possibility that PA6 and SHH/FGF8-based approaches alone are not sufficient to generate authentic midbrain dopaminergic neurons [50, 51]. The field then underwent a period of reevaluation where protocols that recapitulated ventral mesencephalic development with more fidelity and precision were developed. During this time, earlier protocols were systematically refined and superseded by studies using WNT agonists [48, 50], most notably from the Studer Lab who established an efficient midbrain floor plate differentiation strategy through which dopaminergic neurons were efficiently specified. Crucially, this study demonstrated functional engraftment and recovery in mice, rats, and nonhuman primates with Parkinson's disease [50]. Contemporaneous studies showed that by using canonical WNT agonists at different concentrations and for defined durations, the generation of diverse regionally specified progenitors from fore- to hindbrain is possible. Interestingly, the generated midbrain dopaminergic neurons, but not their telencephalic counterparts, could reverse structural and functional deficits in animal models of Parkinson's disease. This subtype specificity highlights the unparalleled potential of* directed* differentiation of hPSCs in regenerative medicine [48]. A further notable study in this arena used transient blockade of FGF signalling to refine midbrain positional identity and yield authentic dopaminergic neurons with high efficiency [52]. Although it can be argued that these later studies yield more authentic midbrain dopaminergic neurons because they utilised developmentally rationalised cues, it should be noted that the GSK3*β* inhibitors such as CHIR99021 used here for WNT pathway activation do have off target effects (i.e., they regulate pathways other than WNT) [53]. Additionally it is noteworthy that more specific WNT pathway activators (e.g., WNT3a) do not reproducibly generate midbrain dopaminergic neurons with the same efficiency as the GSK3*β* inhibitor CHIR99021 [50, 54]. In future studies, the absolute requirement for GSK3*β* inhibition and the identification of additional key regulatory pathways would be of great importance to establish.

### 4.3. Hindbrain and Cerebellum

Broadly, evolutionary pathways appear to be more conserved in caudal (primitive) regions of the CNS such as the hindbrain. The hindbrain can be divided into rostral and caudal portions, which are separated by rhombomere 4 (r4). Neurons derived from rostral regions project to and innervate myriad brain regions, whereas the caudal portion, located in the myelencephalon, gives rise mainly to descending spinal projections. The brain innervating central serotonergic neurons, originating from r2-3 of the rostral raphe, contribute to higher order brain functions and are implicated in a range of psychiatric disorders. By using EGF and FGF2 in the maintenance media, so-called “long-term self-renewing rosette-type” hPSC-derived neural precursors can be expanded which exhibit a ventral anterior hindbrain-like expression profile after prolonged culture [55]. These precursors preferentially generated GABAergic neurons, some of which were serotonergic neurons. This finding likely reflects positional respecification of the default forebrain identity secondary to protracted culture in FGF2, which is known to have caudalising properties. Very recently, a protocol for directed differentiation of hPSCs to functionally validated hindbrain serotonergic neurons through activation of the WNT and SHH pathways was reported [56].

There are few reports of cerebellar differentiation with demonstration of electrophysiologically mature and functional Purkinje- and granule-cell specification [57, 58]. A recent study generated MATH1-positive cerebellar-like granule cells from iPSCs using a complex programme of sequentially administered morphogens, including FGF8, RA, FGF4, FGF2, WNT1a, WNT3a, BMP4, GDF7, BMP7, BMP6, SHH, BDNF, Jagged1, and NT3 [59]. More recently an ontogeny recapitulating strategy for cerebellar neurogenesis achieved efficient directed differentiation of hPSCs using three morphogens only [57]. Here, hPSC-derived embryoid bodies were first positionally specified to the midbrain-hindbrain boundary and subsequently directed to cerebellar plate neuroepithelium (CPNE). CPNE in turn gave rise to functionally mature Purkinje- and granule cells, DCN-neurons, and various interneurons in specific coculture settings by sequentially administering FGF2, FGF19, and SDF1. A contemporaneous study used insulin, FGF2, and an antagonist of SHH signalling (cyclopamine), again necessitating coculture with rat cerebellar slices to reinforce the validity of this approach for directed differentiation to cerebellar neurons [58]. Both of these recent studies relied to some degree on coculture with isotopic organotypic slices/rodent cerebellar derivatives. Future studies in this area should focus on overcoming reliance on coculture with rodent or human cerebellar slice cultures by identifying the requisite extrinsic signals for specifying cerebellar derivatives at each stage of their lineage restriction.

### 4.4. Spinal Cord

The generation of functional spinal cord derivatives, including motor neurons, has been achieved from hPSCs through a variety of approaches using insights from developmental biology [15, 60–62]. These strategies employed either simultaneous or sequential administration of caudalising (e.g., RA) and ventralising (e.g., SHH) morphogens prior to terminal differentiation. These studies confirmed the expression of specific motor neuron fate determining factors including HB9, specific enzymes/transporters including choline acetyltransferase (ChAT) and the vesicular acetylcholine neurotransmitter transporter (vAChT), and also coculture with myotubes to demonstrate the formation of physiologically relevant neuromuscular junctions [18, 60, 63]. Electrophysiological studies confirm that hPSC-derived motor neurons acquire appropriate functional properties [60]. Motor neuron precursors have importantly been shown to survive and integrate in rodent embryonic spinal cord [64, 65] and to project axons forming physiological synapses.

Treating cultures with RA typically results in a cervical or brachial positional identity [18, 65]. More caudal (lumbar) motor neuron fates can also be achieved in the absence of RA signalling, likely in response to FGF2; indeed we have reported a retinoid independent strategy for motor neurogenesis from hPSCs that yields a lumbar spinal subtype identity and favours medial motor columnar specification [18]. This retinoid-mediated diversification of motor neuron subtypes was further supported by a parallel study using mouse embryonic stem cells [65]. A recent study employed combined retinoic acid and WNT agonism to generate cranial motor neurons from hPSCs [66]. Yet another subsequent study reported the derivation of motor neurons under RA treated but SHH free conditions, uncovering important insights into human motor neurodevelopmental biology [67].

### 4.5. Neural Crest

Neural crest cells are highly migratory and give rise to myriad differentiated cell types including (i) sensory and autonomic neurons and Schwann cells, (ii) chromaffin cells in the adrenal medulla, (iii) melanocytes, and (iv) cranial skeletal and connective tissue components. The fate of the neural crest cells is largely determined by where they migrate to/settle. From an hPSC perspective, striking phenotypic consequences have been demonstrated based on plating density, and this provides a strategy to generate neural crest derivatives. A high plating density favours PAX6 expressing central nervous system precursors while low plating density specified neural crest-like differentiation [16]. Using variations of this approach, stage-specific isolation/differentiation of hPSC-derived neural crest cells has been achieved using a combination of in vitro expansion, directed differentiation via extrinsic signals and cell sorting. For example, serum-free conditions with subsequent bespoke programmes of extrinsic cues can permit specification of Schwann cells, autonomic or sensory neurons, while serum based approaches tend to favour mesenchymal derivatives including adipocytes, osteocytes, chondrocytes, and smooth muscle. Functional validation has been demonstrated by transplantation of hPSC-derived neural crest cells into a chick embryo, where they exhibit preserved neural crest identity in the context of survival, migration, and differentiation [68].

## 5. Concluding Remarks

The unrivalled complexity of the mammalian central nervous system is enabled by a series of progressive and sequential events during embryogenesis. The degree of interconnectedness within the central neuraxis is somewhat surprising given its impressively precise organisation into discrete regions. Evolutionary conservation of developmental processes underlying the organisation of such discrete neural regions becomes increasingly less applicable to more rostral (i.e., evolutionarily “newer”) components, like the forebrain. The hPSC platform is emerging as an important reductionist in vitro system to interrogate aspects of human development, which have remained experimentally inaccessible until now.

Current approaches towards such directed differentiation of hPSCs often fail to capture the dynamic and overlapping nature of neurodevelopmental processes. For instance, neural induction and patterning are often conceptualised as mechanistically distinct processes. However, a bias towards different regional fates will likely be determined by the neural conversion paradigm employed. Similarly current differentiation strategies do not yet fully acknowledge or exploit the ability to influence cell (subtype) fate decisions* postmitotically*, which has been reported [69–71]. As such, the field's approach to directed differentiation to individual cellular subtypes could potentially benefit from being more closely aligned to each respective stage of neurodevelopment, leading to bespoke conditions for each stage of lineage restriction (i.e., neural conversion, patterning, and terminal differentiation).

Developmental principles are a crucial resource for defining ontogeny recapitulating directed differentiation protocols for hPSCs (Figure 5). In addition to the wealth of knowledge that already exists from rodent developmental biology, there is an increasing number of publicly available human brain region-specific and transcriptome-wide datasets from studies using a diverse range of tissue from fetal through to adult stages [72–74]. In addition to highlighting the maturational status of hPSC-derived neurons [75], such developmental/stage-specific data sets could now serve as a gold standard for validating directed differentiation protocols to region-specific cell types. Indeed these datasets should eventually contribute to experimental design when a relatively unexplored region of the nervous system is being investigated using hPSCs. The utilisation of human brain-derived data bypasses potential issues of evolutionary divergence between mouse and man, especially in the more rostral (evolutionarily newer) regions of the neuraxis. Coupling insights gained from these invaluable resources together with high throughput platforms for protocol discovery would be a future avenue for improving the robustness of current directed differentiation strategies [66].

Finally, the hPSC field stands to benefit from defining multiple directed differentiation protocols that employ closely aligned methods for neural conversion and similar protocol durations. This may then permit more meaningful comparison between region-specific neurons, without the potentially confounding issue of differential cellular maturation. Indeed such an approach was recently utilised to show region-specific phenotypes using iPSCs derived from patients with Alzheimer's disease and motor neuron disease [76]. Taken together, such standardizations in directed differentiation of hPSCs may help to drive the identification of robust strategies to specify enriched populations of all clinically relevant region-specific subpopulations of human neurons for further study.

## Figures and Tables

**Table 1:** 

Cell type	Study	Culture method	Programme of developmental cues for neural conversion and patterning	Duration (days)
Cortical precursors	Watanabe et al. 2007 [44]	Serum-free embryoid body-like (SFEB)	BMP antagonist (BMPRIA-Fc) Activin/Nodal antagonist (LeftyA) Wnt antagonist (Dkk1)	35

Cortical neurons	Eiraku et al. 2008 [45]	SFEB derivative	BMP antagonist (BMPRIA-Fc) Activin/Nodal antagonist (LeftyA) Wnt antagonist (Dkk1)	45–60

Cortical neurons	Chambers et al. 2009 [16]	Monolayer	BMP antagonist (NOGGIN) Activin/Nodal antagonist (SB431542)	19

Cortical neurons and MGE/LGE neurons	Li et al. 2009 [47]	Suspension	None for cortical (endogenous Wnt) For MGE and LGE derivatives: Wnt antagonist (Dkk1) Sonic hedgehog (SHH)	30–35

Cortical neurons	Shi et al. 2012 [46]	Monolayer	BMP antagonist (NOGGIN) Activin/Nodal antagonist (SB431542)	80–100

Midbrain dopaminergic neurons	Kriks et al. 2011 [50]	Monolayer	BMP antagonist (NOGGIN or LDN) Activin/Nodal antagonist (SB431542) Sonic hedgehog (SHH and purmorphamine), Fibroblast Growth Factor 8b (FGF8b), Wnt agonist (CHIR99021)	80

Midbrain dopaminergic neurons	Kirkeby et al. 2012 [48]	Embryoid body	BMP antagonist (NOGGIN) Activin/Nodal antagonist (SB431542) Wnt agonist (CT99021) Sonic hedgehog (SHH-C24II)	35

Midbrain dopaminergic neurons	Jaeger et al. 2011 [52]	Monolayer	BMP antagonist (NOGGIN) Activin/Nodal antagonist (SB431542) FGF/ERK antagonist (PD0325901) Fibroblast Growth Factor 8b (FGF8b), Sonic hedgehog (SHH)	30–35

Cerebellar neurons	Erceg et al. 2012 [59]	Embryoid body	Fibroblast Growth Factors (FGF8, FGF4, and FGF2) Retinoic acid (RA) Wnt agonists (Wnt1, Wnt3a) BMPs (BMP4, BMP6, BMP7, and GDF7) Sonic hedgehog (SHH)	35

Cerebellar neurons	Muguruma et al. 2015 [57]	SFEBq	Activin/Nodal antagonist (SB431542) Fibroblast Growth Factors (FGF2, FGF19) Insulin Stromal cell-derived factor 1 (SDF-1) (co-culture with mouse granule cells to generate Purkinje cells)	35–135

Cerebellar neurons	Wang et al. 2015 [58]	Embryoid body	Fibroblast growth factor (FGF2) Insulin Sonic hedgehog antagonist (cyclopamine) (coculture with rat organotypic cerebellar slice to generate Purkinje cells)	20–65

Spinal cord motor neurons	Li et al. 2005 [63]	Monolayer	Retinoic acid (RA) Sonic hedgehog (SHH) Fibroblast Growth Factor (FGF2)	21–35

Spinal cord motor neurons	Patani et al. 2011 [18]	Suspension	Activin/Nodal antagonist (SB431542) Sonic hedgehog (purmorphamine) Fibroblast Growth Factor (FGF2)	21–35

Spinal cord motor neurons	Calder et al. 2015 [67]	Monolayer	Activin/Nodal antagonist (SB431542) BMP antagonist (LDN193189) Retinoic acid (RA)	35–40
